# Correlation of skull morphology and bite force in a bird-eating bat (*Ia io*; Vespertilionidae)

**DOI:** 10.1186/s12983-020-00354-0

**Published:** 2020-03-19

**Authors:** Biye Shi, Yuze Wang, Lixin Gong, Yang Chang, Tong Liu, Xin Zhao, Aiqing Lin, Jiang Feng, Tinglei Jiang

**Affiliations:** 1 Jilin Provincial Key Laboratory of Animal Resource Conservation and Utilization, Northeast Normal University, 2555 Jingyue Street, Changchun, 130117 China; 2 Key Laboratory of Vegetation Ecology of Education Ministry, Institute of Grassland Science, Northeast Normal University, 2555 Jingyue Street, Changchun, 130117 China; 3 College of Life Science, Jilin Agricultural University, 2888 Xincheng Street, Changchun, 130118 China

**Keywords:** 3D geometric morphometrics, Bird-eating bats, Skull, Bite force, Diets, Phylogeny

## Abstract

**Background:**

Genetic and ecological factors influence morphology, and morphology is compatible with function. The morphology and bite performance of skulls of bats show a number of characteristic feeding adaptations. The great evening bat, *Ia io* (Thomas, 1902), eats both insects and birds (Thabah et al. J Mammal 88: 728-735, 2007), and as such, it is considered to represent a case of dietary niche expansion from insects to birds. How the skull morphology or bite force in *I. io* are related to the expanded diet (that is, birds) remains unknown. We used three-dimensional (3D) geometry of the skulls and measurements of bite force and diets from *I. io* and 13 other species of sympatric or closely related bat species to investigate the characteristics and the correlation of skull morphology and bite force to diets.

**Results:**

Significant differences in skull morphology and bite force among species and diets were observed in this study. Similar to the carnivorous bats, bird-eaters (*I. io*) differed significantly from insectivorous bats; *I. io* had a larger skull size, taller crania, wider zygomatic arches, shorter but robust mandibles, and larger bite force than the insectivores. The skull morphology of bats was significantly associated with bite force whether controlling for phylogeny or not, but no significant correlations were found between diets and the skulls, or between diets and residual bite force, after controlling for phylogeny.

**Conclusions:**

These results indicated that skull morphology was independent of diet, and phylogeny had a greater impact on skull morphology than diet in these species. The changes in skull size and morphology have led to variation in bite force, and finally different bat species feeding on different foods. In conclusion, *I. io* has a larger skull size, robust mandibles, shortened dentitions, longer coronoid processes, expanded angular processes, low condyles, and taller cranial sagittal crests, and wider zygomatic arches that provide this species with mechanical advantages; their greater bite force may help them use larger and hard-bodied birds as a dietary component.

## Background

Ontogeny, evolutionary history, and ecology (e.g., biological interactions with environmental variables) though selection directly or indirectly influence the morphological variation of organisms [1–3]. Ecomorphology assumes that morphological changes of animals are related to behavior, performance, and fitness; they are influenced by heredity, living environment, and feeding habits [4–8]. Among these factors, feeding habits are the main forces affecting the skull morphological differences of animals [9–12]. Animals with different feeding habits have evolved morphological characteristics related to their diets. These morphological differences are partly reflected in bone structure and body size [13]. Additionally, the evolutionary history of organisms also influences morphology and causes closely related species to exhibit similar morphology [14–16].

The skulls of animals are the most important structures used for perceiving and interacting with the external environment; skulls play an important role in food collection and processing, drinking, vocalization, and respiration [17, 18]. The variation in skull morphology in various bat species are related to feeding ecology in bats [19, 20], different food types are matched by the morphological and functional cranial specializations [21], and thus, skull characteristics can be used to predict phylogenetic relationships [22], food composition, and feeding niche breadth.

Bite force is the basis of numerous types of functional demands of vertebrates (e.g., feeding, mating, defense, and competition) and is closely related to body size and skull morphology [20, 23, 24]. Bite force is also an important performance trait. Increased bite force can broaden the spectrum of available prey [7], and the difference in bite force among various taxa is linked to skull shape divergence and a variety of ecological challenges, such as food acquisition, mating, and predator avoidance [25–28].

The relationships between skull morphology, bite force, and diet have been investigated in a variety of animal groups, including fish [29, 30], lizards [24, 31], crocodilians [32], turtles [33], rodents [12, 34], birds [23]. Many studies have also focused on bats [19–21, 35–40], but studies involving skull morphology, in vivo bite force, and diets in a single study are limited. For example, Nogueira et al. [28] investigated the relationship between skull morphology, bite force, and diet of 14 species in Phyllostomidae and found that the bite force was associated with the former two factors. *Micronycteris microtis*, a carnivorous bat, has a strong mechanical advantage in skull morphology, and thus can generate a sufficient bite force to prey upon lizards [40].

Bats are nocturnal mammals with numerous species. Their feeding systems have a relatively simple anatomy and various species have a highly diverse skull morphology. The effect of natural selection on the skull morphology seems to translate into changes in bite force [28, 38]. The study of ecological adaptation of skull morphology and bite force is useful for revealing the evolutionary trends associated with diet in bats. According to the type of food, bats can be classified as Insectivores, Carnivores, Piscivores, Frugivores, Nectarivores, and Sanguivores [37, 41]. Bats with different feeding habits have different skull morphology and bite force [20]. Currently, a growing number of studies have analyzed the three-dimensional geometrics of bat skull morphology and have taken field measurements of bite force, but most of these studies have been done independently.

Like piscivorous bats [42], bird-eating bats fill a rare dietary niche that has expanded from low- to high-quality food resources. Among the 1300 bat species, *Nyctalus lasiopterus*, *N. aviator*, *Vampyrum spectrum*, and *I. io* are known to regularly consume birds in addition to insects [43–47]. The great evening bat, *I. io*, is widely distributed in southern Asia and is also found in southern China. The species mainly feeds on insects and has been speculated to prey on small passerine birds. We have confirmed that 22 bird species (about 6.3–18.5 g) from seven families of Passeriformes were hunted by *I. io* based on fecal DNA analysis, suggesting a very high prey (birds) diversity (unpublished data, L. Gong). The expansion of the dietary niche enables *I. io* to use more resources in the environment. Their successful predation on birds is inevitably affected by ecological selection. Under such pressures, it is unclear whether the skull morphology of bird-eating bats has undergone certain changes to generate higher bite performance to enable the bats to capture and kill birds.

In this study, we used 3D geometry of the skulls as well as bite force and diet measurements from *I. io* and 13 other sympatric or closely related species to investigate the characteristics and the correlation of skull morphology and bite force to bat diets in a phylogenetic context. We hypothesized that the skull shape and size of *I. io* would be more similar to those of carnivorous bats rather than insectivorous or piscivorous bats. We predicted that *I. io* skulls would have higher sagittal crests, wider and stronger zygomatic arches, shortened jaws and rostrums, taller dentaries, higher coronoid processes, lower condyles, and expanded angular processes. We also hypothesized that *I. io* would have the largest size-specific bite force, since they can capture and kill larger and harder prey (birds) than insectivorous bats. Finally, we hypothesized that skull morphology, bite force, and diet would correlate to each other in the 14 bat species. We predicted that the skull morphology and bite force would be associated with diets, and that the higher bite force would also be related to the specific skull morphology, such as robust mandibles, taller skulls, and wider zygomatic arches.

## Results

### Skull morphology differences among species

**Fig. 1:**
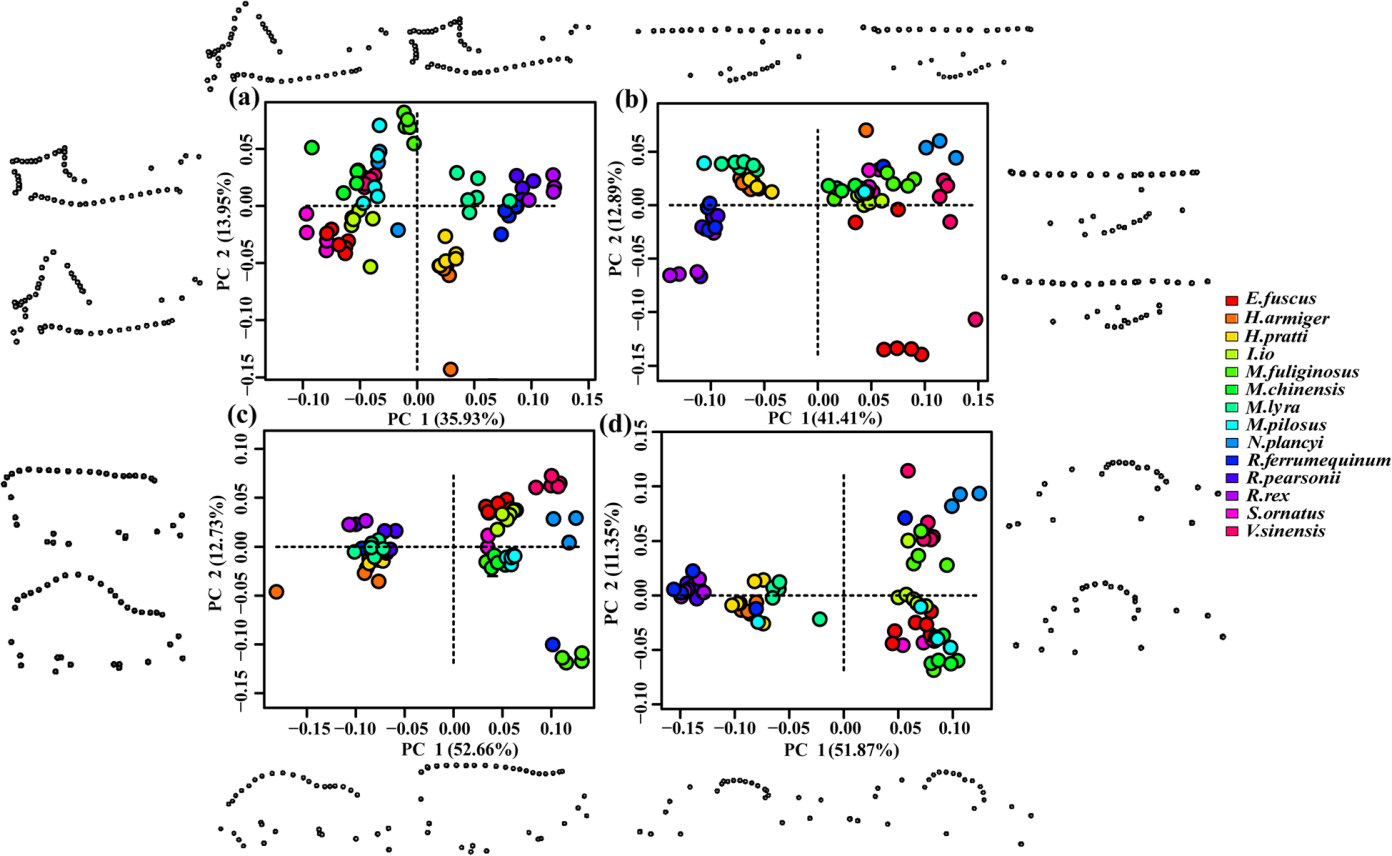
Distribution of 14 bat species according to the first two principal component (PC1, PC2) scores. **a** Mandibular; **b** Dorsal cranium; **c** Lateral cranium; **d** Ventral cranium

Principal component analysis (PCA) showed that different species occupy different positions in tangential spaces. For the mandibular morphology, the first four PCs explained 68.25% of the total variance (PC1 = 35.93%, PC2 = 13.95%, PC3 = 12.27%, PC4 = 6.1%). *Ia io*, *Scotomanes ornatus*, *Eptesicus fuscu*, *Nyctalus plancyi, Vespertilio sinensis, Myotis chinensis*, *Myotis pilosus*, and *Miniopterus fuliginosus* were all located on the same end of the PC axis, and have similar mandible shapes. The skulls of these eight species have shorter and stronger mandibles, taller and wider coronoid processes, lower condyles, and expanded angular processes when compared with the skulls of other species (Fig. 1a; Fig. S1a, Additional file 1). Such interspecific differences also existed in cranial morphology. (i) Dorsal cranium: the first four PCs explained 69.81% of the total variance (PC1 = 41.41%, PC2 = 12.89%, PC3 = 8.55%, PC4 = 6.96%). *Ia io* and its closely related species (*S. ornatus*, *E. fuscu*, *V. sinensis*, *N. plancyi*) were located on the positive end of the PC axis, with shorter crania, wider zygomatics, and wider interorbital spacing (Fig. 1b; Fig. S1b, Additional file 1). (ii) Lateral cranium: the first four PCs explained 82.52% of the total variance (PC1 = 52.66%, PC2 = 12.73%, PC3 = 9.21%, PC4 = 7.92%). *Ia io* and its related species were located on the positive end of the PC axis; the skulls of these species possess shortened heads and rostras, high sagittal crests, and convex supraoccipital bones (Fig. 1c; Fig. S1c, Additional file 1). (iii) Ventral cranium: the first four PCs explained 78.13% of the total variation (PC1 = 51.87%, PC2 = 11.35%, PC3 = 7.76%, PC4 = 7.15%). Skulls of *I. io* also and those of its relatives have wider zygomatic arches and posterior occipitals (Fig. 1d; Fig. S1d, Additional file 1). In summary, both cranial and mandibular characteristics separated the species well; both measures showed that the skull morphology of *I. io* is similar to their closely related species, but were very different from less closely related species (e.g., species in Hipposideridae and Rhinolophidae).

### Skull morphology differences among species with different diets

The results of skull morphological disparity showed that there were no significant differences among dietary groups, whether accounting for phylogeny or not (almost all *P* > 0.05; see Table S1, Additional file 2). However, Procrustes analysis of variance showed that significant differences in cranial and mandibular morphology exists among bat species with different diets (dorsal: *R*^2^ = 0.104, *F* = 2.51, df = 3, *P* = 0.002; lateral: *R*^2^ = 0.112, *F* = 2.73, df = 3, *P* = 0.006; ventral: *R*^2^ = 0.116, *F* = 2.831, df = 3, *P* = 0.003; mandible: *R*^2^ = 0.121, *F* = 2.975, df = 3, *P* = 0.001).

**Fig. 2:**
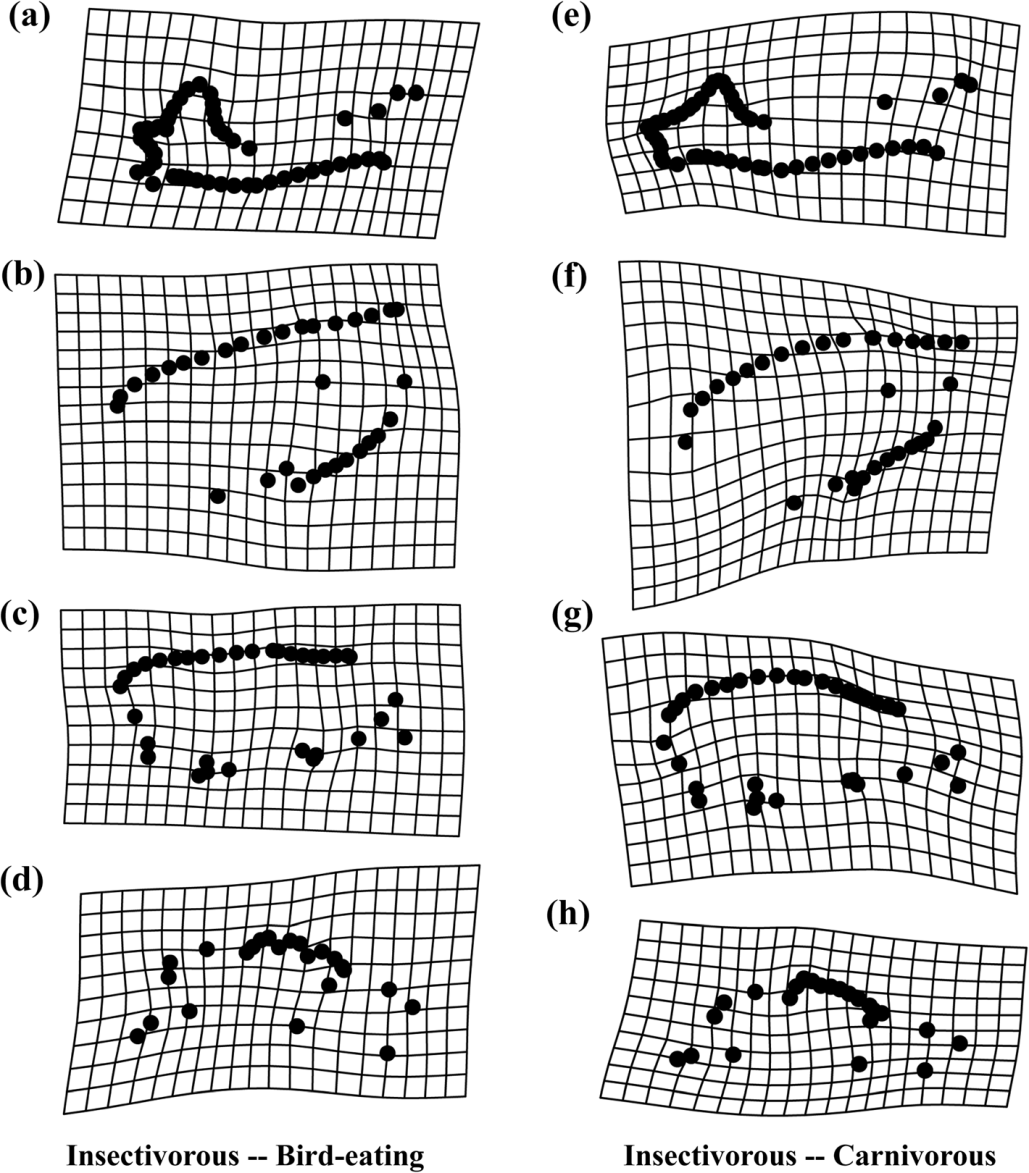
Mesh deformation diagram showing skull shape differences between the mean shape of (**a**, **b**, **c**, **d**) insectivorous and bird-eating bats and (**e**, **f**, **g**, **h**) with carnivorous bats. **a**, **e** Mandible; **b**, **f** Dorsal cranium; **c**, **g** Lateral cranium; **d**, **h** Ventral cranium

The deformation grid of the thin-plate spline showed the differences in skull shape between the bird-eating and insectivorous bats (Fig. 2a, b, c, d). Compared with the insectivores, the bird-eaters displayed shorter mandibles, higher coronal processes, lower and sunken inward condyle processes, expanded angular processes, and increased distance between the last molar and the ascending ramus (Fig. 2a). Carnivorous bats exhibited similar characteristics to the insectivores in addition to the angular processes (Fig. 2e). In the dorsal cranium, both bird-eaters and carnivores had narrower interorbital spacings and wider zygomatic arches than insectivores (Fig. 2b, f). In addition, the bird-eaters and insectivores also differed in the lateral and ventral cranium; the bird-eaters had a convex supraoccipital bone, a shorter dentition and cranial length, and a wider zygomatic arch (Fig. 2c, d). On the whole, the length and width of the cranium, interorbital spacing and dentition length of the bird-eaters were similar to those of the carnivores but were different from the insectivores.

### Skull size and head dimension differences among species with different diets

**Fig. 3:**
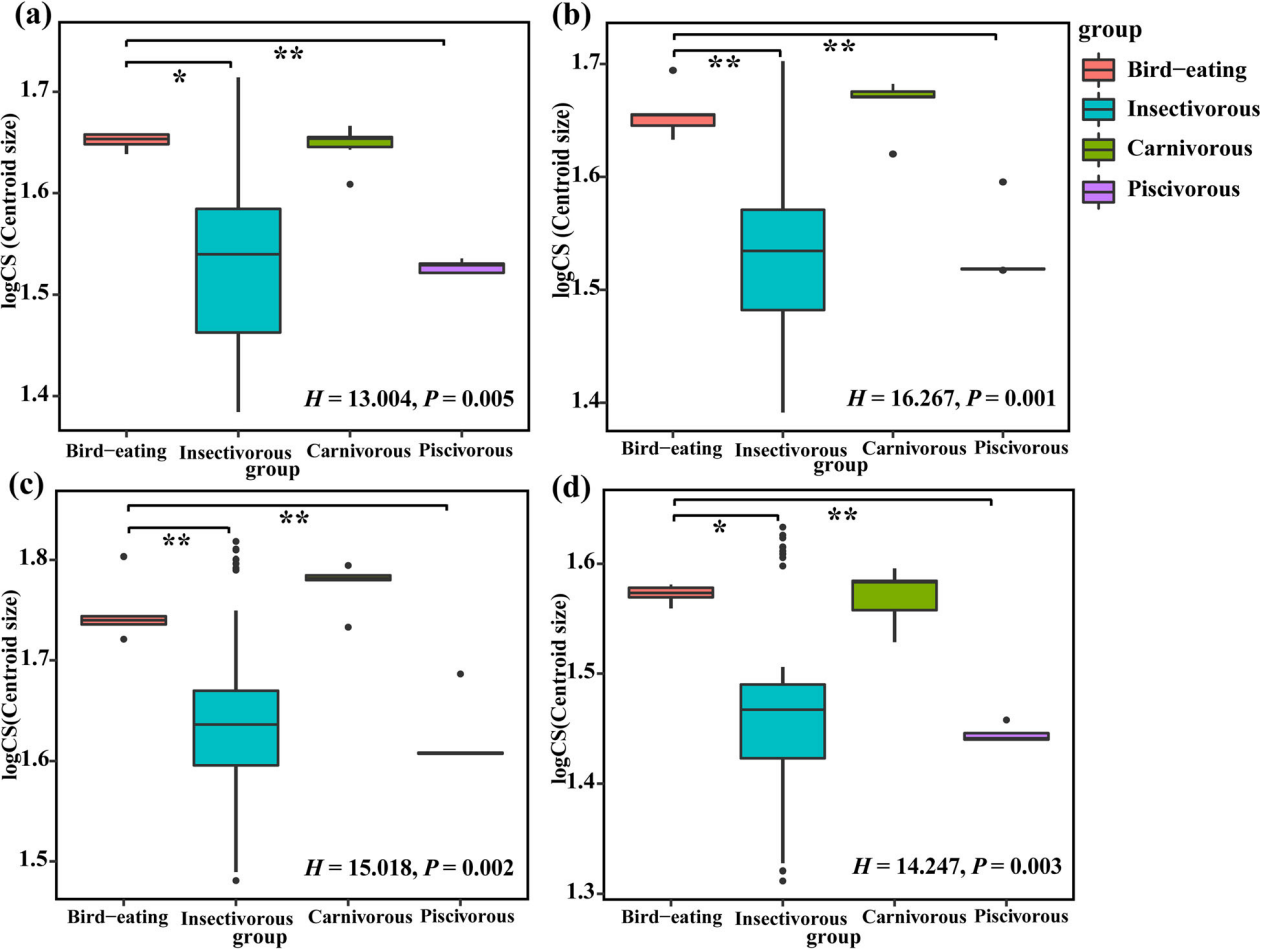
Box plot showing differences in natural log-transformed centroid size (logCS) between four diet categories. **a** Mandible; **b** Dorsal cranium; **c** Lateral cranium; **d** Ventral cranium

The Kruskal-Wallis test showed that the centroid sizes of each part of the skull were significantly different among the bats with four types of diets (all *P* < 0.01). The bird-eating bats had a significantly larger centroid size when compared with the insectivorous and piscivorous bats (all *P* < 0.05, Fig. 3), but no significant differences existed between bird-eating and carnivorous bats in mandibles or between characteristics of the dorsal, lateral, or ventral cranium (all *P* > 0.05, Fig. 3). Furthermore, the Kruskal-Wallis test of the in vivo measurements of external head dimension also showed that significant differences existed in the head length, width, and height among the bats with these four types of diets (all *P* < 0.01). The external head size of bird-eating bats was larger, which was not significantly different from that of carnivorous bats (all *P* > 0.05; see Fig. S2, Additional file 3), but was significantly different from those of insectivorous and piscivorous bats (all *P* < 0.01; see Fig. S2, Additional file 3).

### Bite force differences among species with different diets

The results showed that the bite force was significantly, positively correlated with body size (see Fig. S3, Additional file 4). After correcting for body size, we found that the bite force was significantly different among the four dietary categories (all *P* < 0.05).

**Fig. 4:**
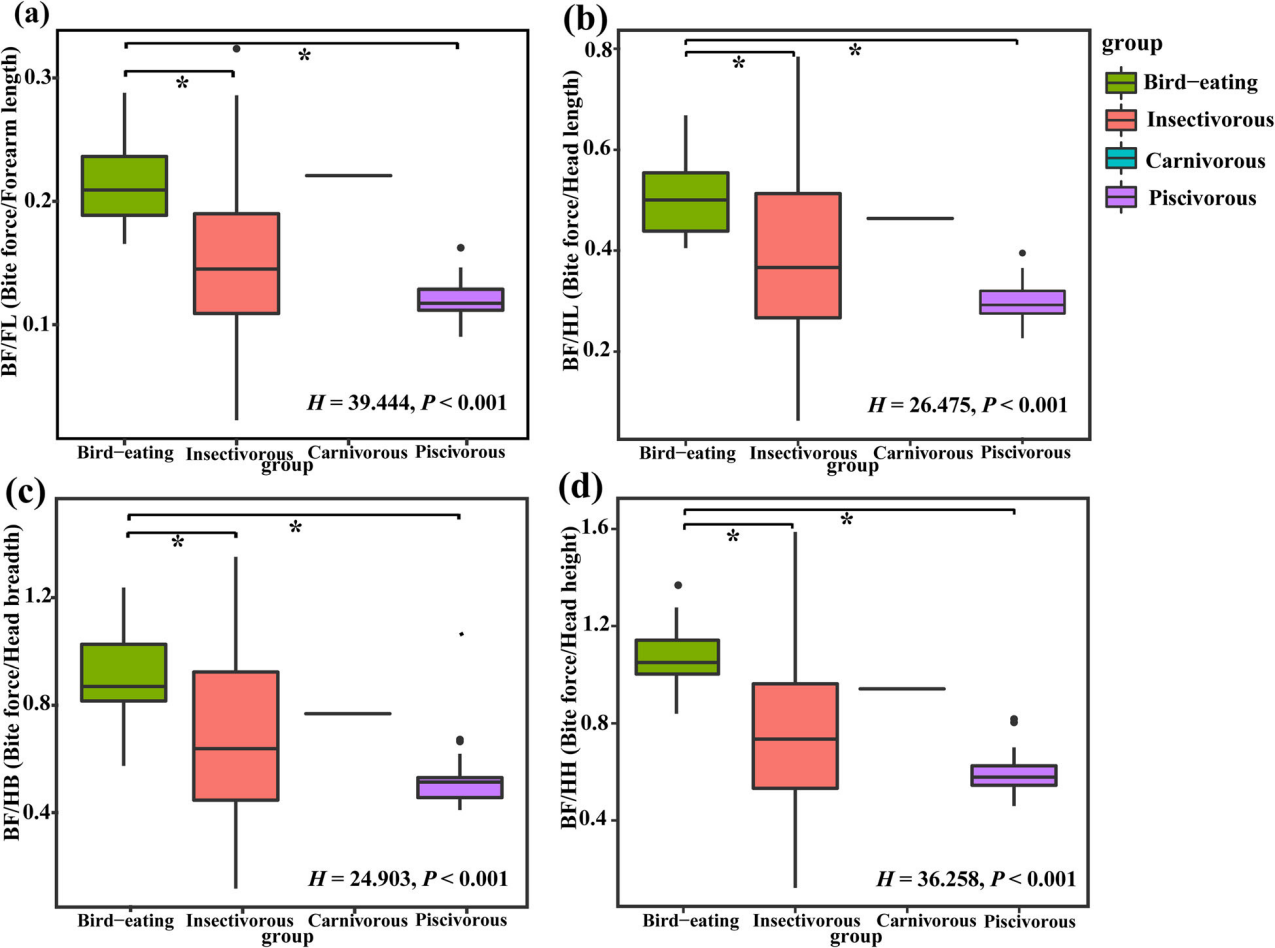
Differences in relative bite force among four diet categories. **a** Corrected forearm length (bite force/forearm length); **b** corrected head length (bite force/head length); **c** corrected head breadth (bite force/head breadth); **d** corrected head height (bite force/head height)

The bite force of bird-eating bats was the largest, followed by that of carnivorous bats, while the piscivorous and most insectivorous bats had relatively small bite forces. The differences in bite force between bird-eaters and insectivores/piscivores were significant (all *P* < 0.001) but were not significant between bird-eaters and carnivores (Fig. 4).

### Relationships among skull morphology, bite force, and diets

Phylogenetic generalized least squares regression (PGLS) showed that the dorsal, lateral, ventral, and mandible of the skull had no allometric effects (all *P* > 0.05). Additionally, the results of PGLS also showed that the skull and residual bite force had no significant correlations with diets (all *P* > 0.05, Table 1).

**Table 1:** The relationships between skull morphology, residual bite force, and diet after controlling for phylogeny

	SS	MS	Rsq	F	Z	P
**Cranium**						
**Dorsal**	0.102	0.034	0.139	0.536	−1.334	1^**a**^
**Lateral**	0.059	0.02	0.104	0.388	−1.682	1^**a**^
**Ventral**	0.085	0.028	0.1	0.37	−1.49	1^**a**^
**Mandible**	0.093	0.031	0.177	0.717	−0.714	0.764
**Residual Bite Force**	0.761	0.254	0.309	1.488	0.625	0.296

^a^Bonferroni correction

Significant correlations were observed between all parts of the skull and residual bite force (all *P* < 0.05) when phylogeny was taken into account (Table 2). After accounting for phylogeny, the relationships between ventral cranium and mandible skull morphology with bite force were still significant (Table 2). Therefore, the skull morphology of bats was significantly associated with residual bite force whether or not phylogeny was controlled for.

**Table 2:** The relationship between skull (cranium, mandible) morphology and the bite force with or without phylogenetic effects. Bold fonts indicates significant differences (*P* < 0.05)

Location	SS	MS	Rsq	F	Z	P
**Dorsal cranium**						
Phylogeny	0.045	0.045	0.301	5.163	2.774	**0.018**^**a**^
Non-phylogeny	0.084	0.084	0.114	1.548	1.05	0.504^a^
**Lateral cranium**						
Phylogeny	0.029	0.029	0.215	3.293	1.872	0.111^a^
Non-phylogeny	0.043	0.043	0.075	0.976	0.23	1^a^
**Ventral cranium**						
Phylogeny	0.069	0.069	0.352	6.524	2.672	**0.009**^**a**^
Non-phylogeny	0.172	0.172	0.203	3.053	2.03	**0.042**^**a**^
**Mandible**						
Phylogeny	0.044	0.044	0.416	8.522	3.577	**0.001**
Non-phylogeny	0.105	0.105	0.201	3.012	2.61	**0.002**

^a^Bonferroni correction

**Fig. 5:**
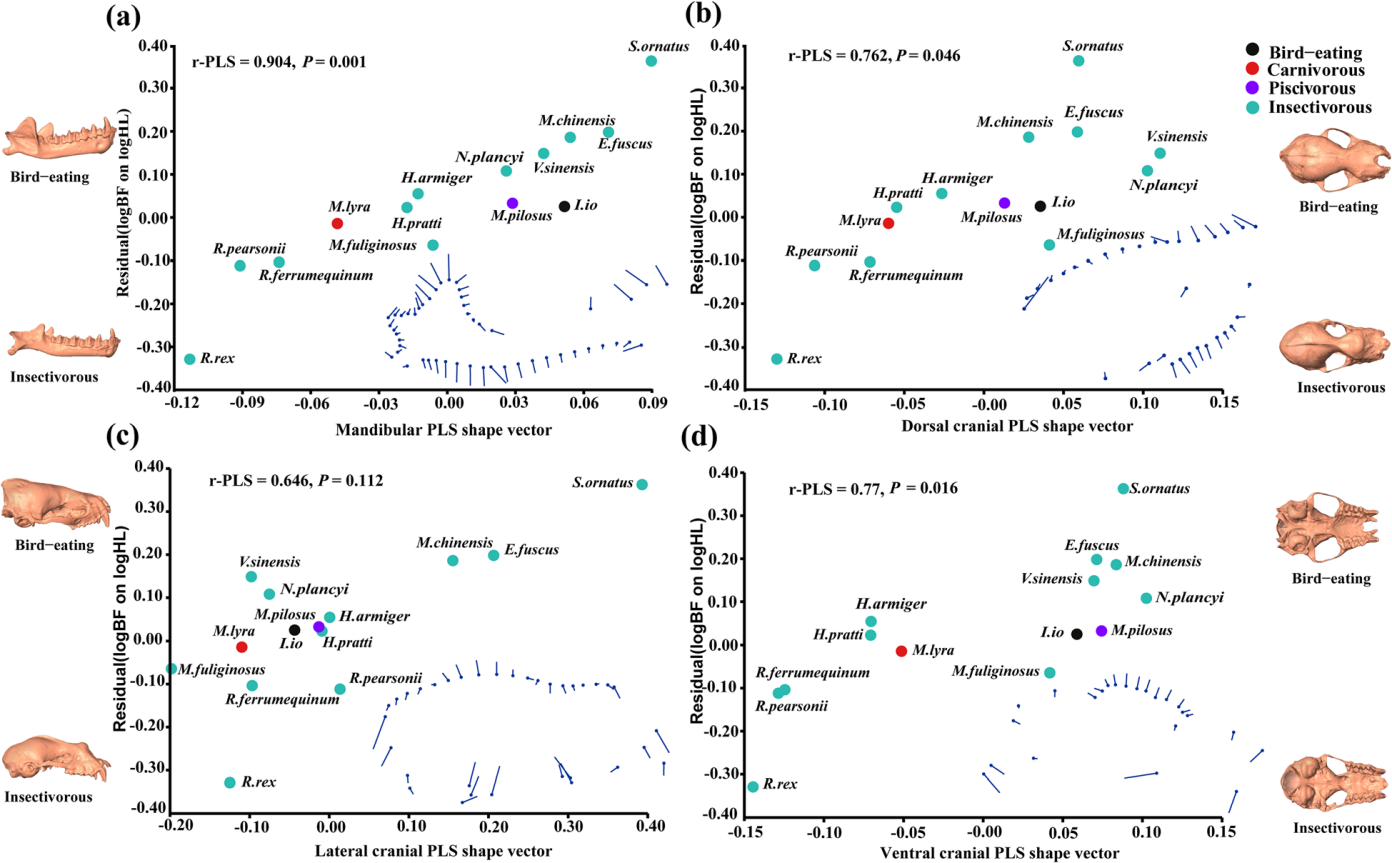
The regression of the residual bite force on the skull partial least squares (PLS) shape vector. **a** Mandible; **b** Dorsal cranium; **c** Lateral cranium; **d** Ventral cranium. The bottom is the change in shape. Above, the skulls of a bird-eating bat (*Ia io*). Below, the skulls of an insectivorous bat (*Rhinolophus ferrumequinum*)

Two-block partial least squares (PLS) results showed that the residual bite force was significantly correlated with the morphology of the dorsal cranium, ventral cranium, and the mandible (The correlation coefficient between two blocks: r-PLS were 0.762, 0.77, 0.904, respectively; all *P* < 0.05) except for the lateral cranium (r-PLS: 0.646; *P* = 0.112). Among these, bite force was most affected by the mandible, i.e., a larger bite force was often accompanied by a stronger mandible, shorter dentition, a taller coronoid process, a lower condyle, and an expanded angular process (Fig. 5a). Moreover, greater bite force was also accompanied by a relatively tall sagittal crest, a convex supraoccipital bone, a shortened head length, and a widened zygomatic arch (Fig. 5b, c, d). Compared with the insectivorous bats, the bird-eating bats of *I. io* displayed stronger jaws with taller and thicker dentaries, shortened dentition, a taller coronoid process, an expanded angular process, a lower condyle, higher cranial sagittal crests, and wider faces and zygomatic arches, which produced a relatively high bite force (Fig. 5).

### Phylogenetic signals

**Fig. 6:**
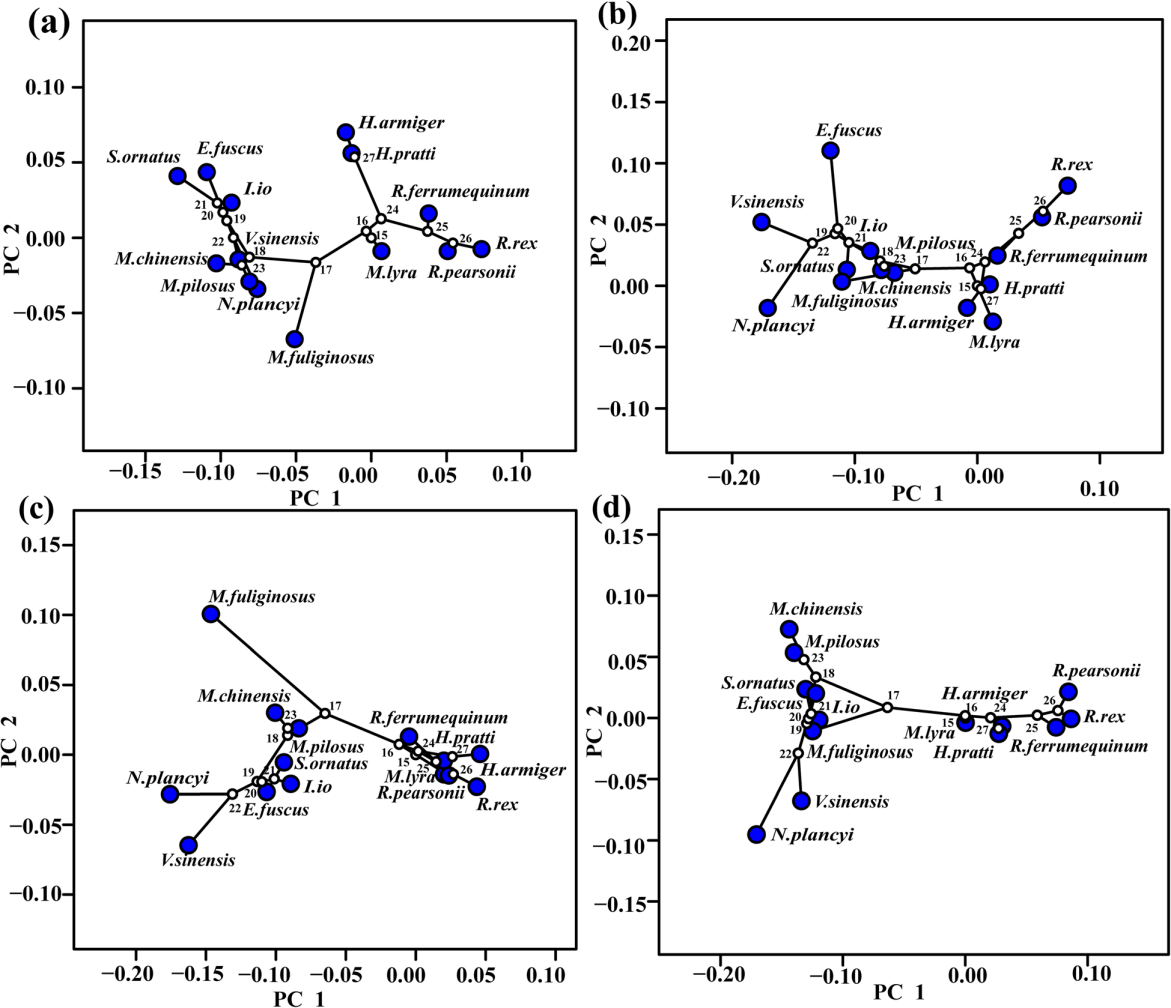
Phylomorphospaces (Principle component (PC) 1 versus PC2) of the skull mean shape in these species. **a** Mandible; **b** Dorsal cranium; **c** Lateral cranium; **d** Ventral cranium

Our results revealed that strong phylogenetic signals in skull shape were detected, except for the regression residual of the ventral cranium on size corrected bite force (K = 0.471, *P* = 0.087; see Table 3). In addition, the phylogenetic signals were also present in the dispersion of the species in skull phylomorphospaces (Fig. 6), where closely related species are adjacent in four morphospaces; that is, *I. io* is close to its related species.

**Table 3:** The phylogenetic signal of skull shape based on regression residuals. K, observed phylogenetic signal; P, the significance level of the observed signal. RBF: Phylogenetic signals based on regression residuals of skull morphology and size corrected bite force (Residual bite force); Diet: Phylogenetic signals based on regression residuals of skull morphology and diet. Bold fonts indicates significant differences (*P* < 0.05)

	Dorsal cranium		Lateral cranium		Ventral cranium		Mandible	
	RBF	Diet	RBF	Diet	RBF	Diet	RBF	Diet
**K**	0.569	0.647	0.724	0.727	0.471	0.632	0.474	0.584
**P**	**0.006**	**0.009**	**0.004**	**0.006**	0.087	**0.011**	**0.017**	**0.011**

## Discussion

In this study, we found that the skull size and morphology of *I. io* was significantly different from other species but similar to closely related species and carnivorous bats, displaying higher mechanical advantage than that of other species. These results supported our first hypothesis. Moreover, with the exception of certain hard insect-eating insectivorous species (i.e., *S. ornatus, H.armiger*), *I. io* had a bite force that was stronger than that observed in insectivores and piscivores in general; but was similar to carnivorous bats, supporting our second hypothesis. Additionally, although we found that the skull morphology of bats was significantly associated with bite force whether or not controlling for phylogeny. The skull and residual bite force had no significant correlation with diets after controlling for phylogeny, which may partly support our third hypothesis. These results showed that phylogeny influenced skull morphology and the size of bats, leading to variation in bite force, and finally to feeding on different prey.

### Differences in skull morphology among species

Our results showed that the skull morphology of *I. io* was separated from the insectivores in Hipposideridae and Rhinolophidae (Fig. 1; Fig. S1, Additional file 1), reflecting the differences in foraging behavior and niches between the sympatric species. Because limited habitat or food availability drives inter- and intraspecific competition [48], such morphological differences optimize the use of resources and avoids diet overlap in various species, thus facilitating species coexistence. For example, the morphological differences in four *Artibeus* species affect their food choice and foraging behavior when feeding on fruits, resulting in ecological segregation and coexistence [49].

However, *I. io* and other Vespertilionids show more similarities in skull shape than other groups of bats, especially to their closely related species (e.g., *S. ornatus*, *E. fuscu*, *N. plancyi*, and *V. sinensis*) (Fig. 6), suggesting that phylogenetic relationships played an important role in skull shape evolution of *I. io*. The results also further confirmed that skull shape were affected by phylogeny (Table 3). Our results were consistent with those of previous studies [50–52], which confirmed that the morphology of the cranium or mandible are affected and restricted by phylogeny in birds and mammals.

### Differences in skull morphology among species with different diets

Mounting evidence has suggested that a significant relationship exists between morphological variation and dietary specialization in bats [21, 40]. The skull shape and size of *I. io* greatly differed from those of the insectivorous bats but were less different from the piscivorous *M. pilosus* in this study. Our results confirmed that the bird-eaters (*I. io*) have higher mechanical advantages in their skulls, similar to those of carnivorous bats (*Megaderma lyra*). Such convergence was the result of the adaptation of the feeding apparatus to different diets, because feeding on various prey requires different functional requirements, and such morphological innovations dominate feeding performance and trophic level [39]. Prey size and hardness play an important role in shaping the phenotypes of bats [21, 53]. Bird-eaters and carnivores both feed on larger vertebrates with soft external bodies and hard internal bones, which require a higher mechanical advantage to bear the heavy loads involved in predation and chewing of food. For example, animal-eating species in the Phyllostomidae have relatively convergent cranium and mandible morphology with carnivorous species, but are quite different from the insectivorous species [35]. In *Myotis*, the skull morphology of the facultatively piscivorous species and piscivorous species (*Myotis vivesi*) was quite similar, with both being significantly different from that of the insectivores [54]. Thus, skulls with high mechanical advantages in carnivorous bats including bird-eaters are the main reasons why they are able to prey on terrestrial vertebrates.

### Differences in bite force among species with different diets

In general, among the four categories considered herein, bird-eating bats had the strongest bite force, which was similar to that of carnivorous bats and was significantly stronger than piscivores and most of the insectivores. In this case, bird-eaters and carnivores may have generated a greater bite force than those of piscivores and insectivores to kill and overcome the resistance of their prey (e.g., vertebrates). This is consistent with previous studies on various diets of bats. For example, the frugivorous species possess the largest bite force, while the nectarivorous species have the smallest; the bite force of insectivorous bats falls between these [21, 28]. Also, the few species feeding on hard insects (i.e., *S. ornatus, H. armiger, H. pratti*) had relatively high bite force within the insectivorous group, similar to the previous studies showing that the bite force of *Scotophilus heathi* (hard beetle-eating) was larger than *Rhinolophus luctus* (soft moth-eating) [55]. These results indicate that the variation of bite force in bats may be related to the hardness of food, and that feeding on vertebrates with harder endoskeletons requires greater mechanical force to bite and chew. Similarly, the degree of food hardness a species can handle is limited by the strength of its bite and the loads that skull can withstand [56]. However, the specific relationship between bite force and food hardness in these species remains to be investigated in our future study.

The difference in bite force is also related to the available food resources, because a larger bite force can allow a species to use more diverse food resources in the environment [38]. Many studies have shown that bite force is an important factor in determining the dietary niche of bat species because a low bite force constrains the species to use a limited portion of the available resources [57–61]; in addition, dietary specialization may lead to a decline in the trophic breadth of certain species through affecting bite force [62, 63]. Here, the largest bite force was observed in *I. io*, which may be another reason for its dietary niche expansion from low to high quality prey.

The bite force of animals is also affected by body size [27], with larger-bodied species having a stronger bite force allowing them to consume a broader range of food resources [53, 64]. Interspecific differences in bite force were also related to body size in lizards [65], turtles [64, 66] and bats [38, 67], with larger body and head dimensions accompanied by greater bite force. In our study, bird eaters have larger skull sizes as well as head dimensions (Fig. 3; Fig. S2, Additional file 3); a significant positive correlation was observed between bite force with both the forearm length and head dimensions (Fig. S3, Additional file 4), indicating that the larger body size and head of *I. io* has led to the greater bite force. Our results were consistent with previous studies in bats. For example, the bite force of Phyllostomid bats was positively correlated with body mass, head height, and head length [28, 38]. Senawin et al. [67] also found that the bite force of a diverse group of insectivorous bats was positively correlated with their forearm length, body mass, and head dimensions; larger body and head were accompanied by greater bite force.

### The relationship between skull morphology with bite force and diet

The adaptation of animals to novel trophic niches is often accompanied by extensive skull morphological specializations [68–70]. Thus, specific bite force is not only an adaptation to diet but also a product of evolution of the feeding apparatus. Previous studies on various animals have confirmed that the morphology of the cranium and mandible affect bite force [28, 71, 72]. In sigmodontine rodents, strong biters have a more compact and robust skulls and mandibles, whereas weaker biters are more gracile and elongate skulls [12]. Bite force in horned lizards (*Phrynosoma*) was also associated with morphological changes in jaw and head [73]. Also, in phyllostomid bats, the different bite forces in various species correspond to specific skull structures, where those species with shorter and taller craniums and mandibles possess a stronger bite force [28]. Our results show that the skull morphology of bats was significantly correlated with bite force (Table 2), in agreement with the pattern observed above. The bats of *I. io* have robust mandibles (that is, taller and thicker dentaries), shorter dentitions, higher coronoid processes, expanded angular processes, and low condyles. The cranium has relatively high sagittal crests, convex supraoccipital bones, reduced interorbital spacing, and more robust and wider zygomatic arches (Fig. 5). The fact that these morphological characteristics of the skull are associated with the largest bite force in *I. io* reflect the basic biomechanical properties of the skull. In mandibular lever systems, a shorter jaw has shorter out-lever arms but longer temporalis moment arms; hence, it has a greater mechanical advantage to produce a higher force output [27, 74].

Additionally, muscle types, fiber length and orientation are also important in bite force production by affecting muscle force [75]. In other animals, fiber length may constrain the gape angles at which force can be optimally produced, and changes in fiber orientation has an effect on pennation angle that may either change the bite force and provide an advantage for jaw opening or closing [76]. Temporalis muscle attachment (volume) and physiological cross-sectional area are related to body size and also affect the bite force [75, 77], in that species with a larger cranium, a larger temporalis mass, and shorter temporalis fiber lengths bite harder. Thus, larger head size and the features of the skull in *I. io* such as robust mandibles, high cranial sagittal crests, and wider zygomatic arches allow for more jaw adductors (temporalis) and specific muscle fiber length and orientation, which may lead to elevated bite force to help *I. io* withstand the high biomechanical loads imposed by their prey (i.e., birds). However, only by determining a direct link between mandibular adductors and bite force can this question be resolved.

A large number of studies have shown that a greater bite force is related to changes in the prey spectrum or prey types consumed [31, 57]. Bird-eating in bats may represent a case of dietary niche expansion from low-to high-quality food resources, which may increase fitness. Relative to the other bat species, *I. io* have greater bite force and we also found that hair of *I. io* was smoother and brighter, which may suggest higher fitness. Thus, the bite force of *I. io* may affect the diet, and finally increase the bats’ fitness. Surprisingly, the skull morphology and bite force were independent of diet after controlling for phylogenetic effects (Table 1), which suggested that phylogeny has a greater contribution than diet on the skull morphology and bite force in these bats. These observations may have two explanations. Arbour et al. [78] found that echolocation related to an early lineage of species had a greater contribution than diet to the evolution of the cranium in bats. Moreover, the skull morphology in carnivores and marsupials are strongly influenced by phylogeny, especially in felids, ursids, and canids [26, 79, 80]. In this study, the insectivorous species (such as *S. ornatus*, *E. fuscu*, *V. sinensis*, and *N. plancyi*) that were closely related to *I. io*, tended to be close each other in morphometric space (Fig. 6), which means the average amount of shape change along the branches of the tree is relatively small. Thus, the common evolutionary history may weaken the influence of diet on morphology and bite force among those species. Additionally, here accurate information related to diet and the physical properties of food in many species were lacking, resulting in insectivorous species accounting for a large proportion (78.57%), which may also affect our results. Future research would reveal in depth the relationship between skull morphology, bite force, and diet by more accurately determining the diets of each species and increasing the number of species in each dietary taxon.

## Conclusions

We used three-dimensional geometric morphology methods and a phylogenetic comparison framework to explore the relationships and adaptive features between skull morphology, bite force, and diet of a bird-eating bat (*I. io*). The results showed that when compared to other bat species, *I. io* had a skull providing it with a mechanical advantage including robust mandibles with shortened dentitions, higher coronoid processes, expanded angular processes, low condyles, and large skull size with a higher cranium, convex supraoccipital bones, reduced interorbital spacing, as well as more robust and wider zygomatic arches. This species also had a stronger bite force that with skull characteristics enable this bat species to hunt relatively large and hard birds, similar to *M. lyra* (a carnivorous bat). Moreover, our results suggested that phylogeny had a greater impact on the characteristics of skull morphology than diet and further impacted on the bite force of these species. A critical limitation of the present study was the relatively small number of species and narrow range of diets, especially for bird-eating, piscivorous, and carnivorous bats. This may have weakened the effects of diets on skull morphology because a large proportion of the species studied here were insectivorous. Thus, more bat species and detailed food hardness data would be needed in a future study. Additionally, the foraging strategy of *I. io* may also play an important role in prompting the bats to hunt birds. Thus, further behavioral observations in *I. io* will also help in understanding the adaptation of bats to hunting birds.

## Methods

### Samples and dietary classification

From April to August in 2018, we collected bite force and skull morphology data of 14 species of bats (see Table S2, Additional file 5). These bats were divided into four categories: (i) Insectivorous: *S. ornatus*, *E. fuscu*, *N. plancyi*, *V. sinensis*, *M. fuliginosus*, *M. chinensis*, *Hipposideros armiger*, *Hipposideros pratti*, *Rhinolophus ferrumequinum*, *Rhinolophus pearsonii*, *Rhinolophus rex*; (ii) Carnivorous: *M. lyra*; (iii) Piscivorous: *M. pilosus*; and (iv) Bird-eating: *I. io*. Diets were classified based on the feeding behavior and the literature [37, 81–85].

### Bite force and body size measurements

Bats were captured using mist nets when leaving their roosts or caves. After capture, the age, sex, and reproductive status were evaluated, and only adult males and adult non-pregnant, non-lactating females were used for the measurements. Age and sex were identified based on the degree of ossification of the coat and tibia [86]. Reproductive status in females was determined by examining the nipples and palpitation of the abdomen [87]. Then, bats were put into cloth bags and transferred to the field laboratory to measure the bite force using a bite force test system (Nanjing Bioinspired Intelligent Technology Co., Ltd., Nanjing, China) with an accuracy of 0.01 N. Bite force was recorded at the molars. According to the methods in Freeman and Lemen [88], measurements were repeated five times for each bat with an inter-trial interval of at least 5 min. The maximum value of the five measures was considered as the maximum bite force produced by that individual. The bite force of the species was calculated by averaging the maximum bite force of each individual. We also measured forearm length, head length, head width, and head height using a digital caliper (TESA-CAL IP67, Tesa Technology, Renens, Switzerland). All of the measurements were taken within 1–2 h of capture, and the bats were released afterward.

### 3D skull morphology data

We used a Blu-ray 3D scanner (OKIO-5 M-100, TianYuan, Beijing, China) to scan every direction of each skull. The scanning accuracy was 0.005 mm with a 0.04 mm sampling point distance. Then, we spliced all of the directional data together by a Geomagic Control X 2018.0.0 (3D Systems., Inc., Rock Hill, SC) to obtain complete three-dimensional skull images. According to the 3D geometric morphology method, 56 three-dimensional landmarks and 83 semi-landmarks from the cranium and mandible were selected to quantify the skull morphology (see Additional file 6 for details). The landmarks were digitized using Landmark Editor 3.6 [89]. Each skull was digitized three times, and the average value was used for subsequent analysis. In order to avoid redundant information related to symmetrical skull structure, we selected the right side of the cranium and mandible for analysis.

For damaged and incomplete skulls, we estimated the location of missing landmarks using the function “estimat.missing” in package “geomorph” [90] in R 3.5.1 [91]. The missing landmarks in the incomplete specimens were designated by NA in place of the x,y,z coordinates. We then used the thin-plate spline (method = “TPS”) to interpolate landmarks on a reference specimen to estimate the locations of missing landmarks on a target specimen. Then, we performed a Generalized procrustes analysis (GPA) for all specimens landmark coordinates with the function “gpagen” in package “geomorph” [90] in R 3.5.1 [91]. The landmarks and semi-landmarks were superimposed in a common coordinate system to remove the effect of location, orientation, and scale among various samples. Here, the Procrustes distance was used to optimize the semi-landmark positions along the curve to obtain the Procrustes aligned coordinates and centroid size. After all specimens’ coordinates had been superimposed using GPA, we obtained the mean shape of each species’ skull from the global object by the function “mshape” in package “geomorph” [90] in R 3.5.1 [91] for subsequent analysis.

### Molecular data and phylogenetic tree

To ensure a relatively accurate reconstruction of the phylogenetic tree, we combined mitochondrial genetic data with the nuclear genetic data. Based on previous studies [92, 93], DNA sequences of 14 species were extracted from wing tissue. The mitochondrial gene Cytb (1140 bp), nuclear genes Chd1 (595 bp), and Acox2 (560 bp) were amplified (see Table S3 for details, Additional file 7). We used BioEdit 7.2.5 [94] to splice and align target sequences, and selected the best fitting alternative model with PartitionFinder 2.1.1 [95]. We constructed a phylogenetic tree for these 14 species using MrBayes 3.2.6 [96] (see Fig. S4, Additional file 8).

### Statistical analysis

Linear regression was performed to assess the effects of body parameters on the changes in bite force. In the models, the averages of the bite force and body size (forearm length, head length, head width, and head height) of each species were used. The Kolmogorov-Smirnov test results showed that our data did not fit a normal distribution, and thus we used nonparametric Kruskal-Wallis tests to compare the relative bite force after correcting for body size (bite force/ forearm length, bite force/head length, bite force/head breadth, bite force/head height), external head dimension (logarithmic transformation of the head length, head width, head height), and skull size (logarithmic transformation of the centroid size) in four dietary categories using 1000 permutation tests. Then, we paired the bird-eating bats with the other three dietary categories for nonparametric pairwise comparisons to explore the differences between the bird-eaters and the other dietary categories.

To explore the differences in skull morphology among different species and diets, we performed PCA on the morphological coordinates of the crania (dorsal, ventral, lateral) and mandibles in all of the individuals using the “plotTangentSpace” function in package “geomorph” [90] in R 3.5.1 [91]. We analyzed the mean shape of the cranium and mandible in four dietary categories (Insectivorous, Carnivorous, Piscivorous, Bird-eating) to obtain the differences in morphology using the “procD.lm” functional in package “geomorph” [90] in R 3.5.1 [91]. To explore the effects of skull size on morphology, we used the mean shape and mean centroid size (logCS) of each species to perform an allometric analysis with the procD.pgls functional in the package “geomorph” [90] in R 3.5.1 [91].

The relationships between bite force, skull shape, and diet were assessed through multiple linear regression and PGLS by the procD.lm and procD.pgls functions in the package “geomorph” [90] in R 3.5.1 [91]. Here, the shape coordinates were considered as the dependent variables; the residual bite force (the regression residual of log bite force on log head length) and diet were considered as independent variables. We employed a two-block PLS analysis in package “geomorph” [90] with function “two.b.pls” to find a linear combination of the shape variables showing maximum covariance with bite force. In this analysis, the mean shape coordinates of the skull were included as independent variables (Block1), and residual bite force was included as a dependent variable (Block2). We used the “morphol.disparity” function in package “geomorph” [90] in R 3.5.1 [91] to compare the disparity in skull morphology among various diets whether or not phylogeny was controlled for. The Procrustes aligned coordinates were used in the function to calculate the Procrustes variance of each group, which depended on the group means, using the residuals from our model. Significance was evaluated by 1000 permutations, where vectors of residuals were randomized among the two groups. We also used the “plotRefToTarget” function in package “geomorph” [90] in R 3.5.1 [91] to visualize the skull differences through deformation grids. As a final step, we evaluated the phylogenetic signals of skull morphology under a Brownian motion model of evolution using the “physignal” function in package “geomorph” [90] in R 3.5.1 [91], which uses the K statistic [97] for highly multivariate data [98]. The phylogenetic signal tests were based on the residuals of the general linear regression model [99]. Here, the mean shape Procrustes coordinates of all species were dependent variables; the independent variable was bite force or diet; and 1000 random permutations were conducted to assess statistical significance. To visualize the variation in shape among species associated with phylogeny, we carried out the function “plotGMPhylomorphoSpace” for the mean shape in package “geomorph” [90] in R 3.5.1 [91].

## Supplementary information

**Additional file 1 : Figure S1.** Distribution of characteristics of 14 bat species according to the first two principal components (PC1, PC3) scores. (a) Mandibular; (b) Dorsal cranium; (c) Lateral cranium; (d) Ventral cranium.

**Additional file 2 : Table S1.** Paired comparison of skull morphological disparity with and without phylogenetic effects.

**Additional file 3 : Figure S2.** Box plot showing differences in natural log-transformed head dimensions between four diet categories. (a) Head length; (b) Head breadth; (c) Head height.

**Additional file 4 : Figure S3.** Regression of the logarithm of bite force against the body size.

**Additional file 5 : Table S2.** Species, sample size (N), diets, and collection locations of bats.

**Additional file 6 :** Description of the cranial and mandibular landmarks and semi-landmarks.

**Additional file 7 : Table S3.** Information related to genes used in the study.

**Additional file 8 : Figure S4.** Phylogenetic relationships among 14 species.

## Data Availability

The datasets generated during and/or analyzed during the current study are available from the corresponding author on reasonable request.

## Abbreviations

PCA: Principal component analysis
PC: Principal component
PGLS: Phylogenetic generalized least squares regression
PLS: Partial least squares
GPA: Generalized procrustes analysis
