# Supplementary figures and images for: Correlation of skull morphology and bite force in a bird-eating bat (Ia io; Vespertilionidae)
Source: Front Zool. 2020 Mar 19;17:8. doi: 10.1186/s12983-020-00354-0 (PMC7082990; doi:10.1186/s12983-020-00354-0)

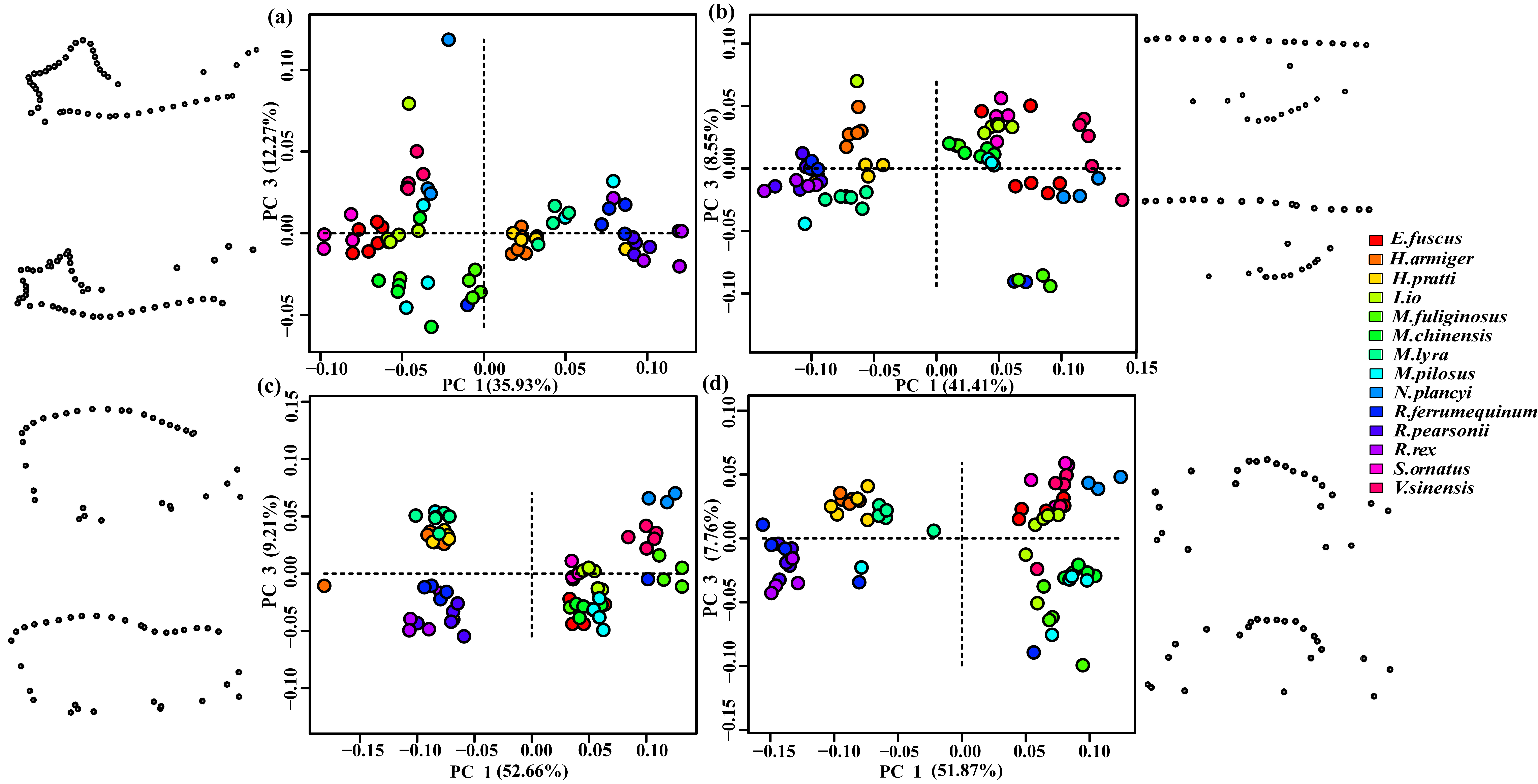

Supplement: Supplementary file 1 — Additional file 1 : Figure S1. Distribution of characteristics of 14 bat species according to the first two principal components (PC1, PC3) scores. (a) Mandibular; (b) Dorsal cranium; (c) Lateral cranium; (d) Ventral cranium. [file 12983_2020_354_MOESM1_ESM.tif]

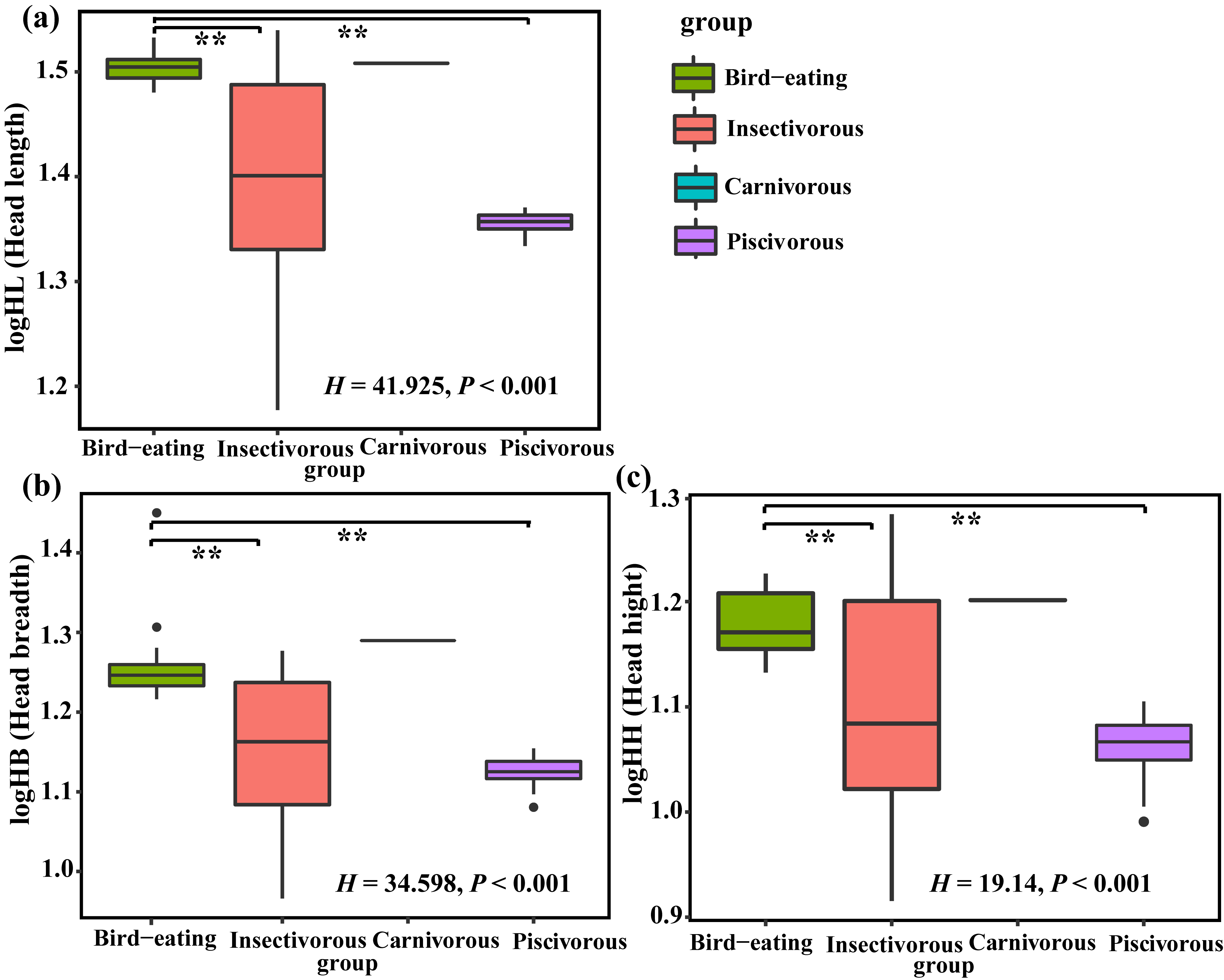

Supplement: Supplementary file 3 — Additional file 3 : Figure S2. Box plot showing differences in natural log-transformed head dimensions between four diet categories. (a) Head length; (b) Head breadth; (c) Head height. [file 12983_2020_354_MOESM3_ESM.tif]

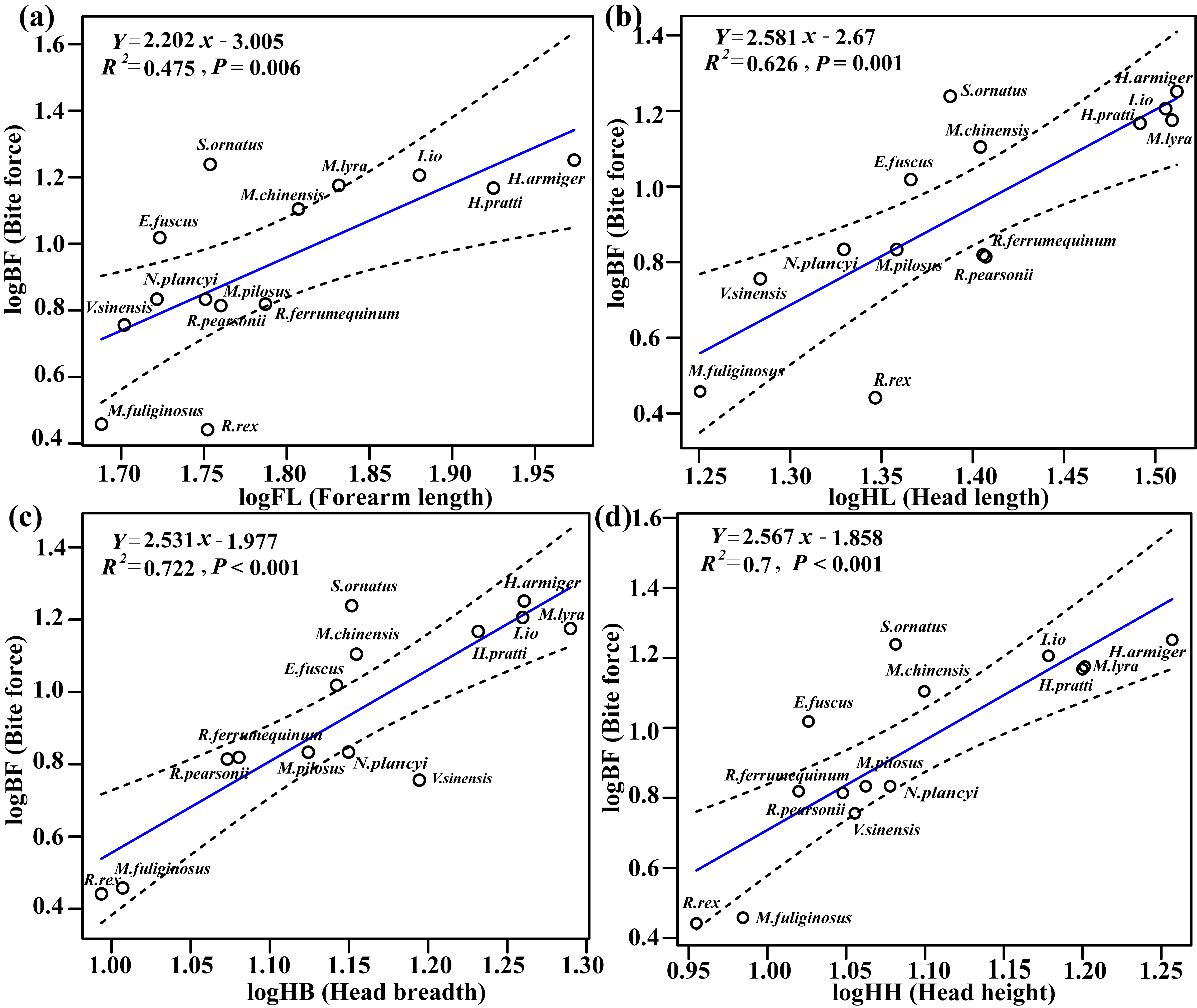

Supplement: Supplementary file 4 — Additional file 4 : Figure S3. Regression of the logarithm of bite force against the body size. [file 12983_2020_354_MOESM4_ESM.tif]

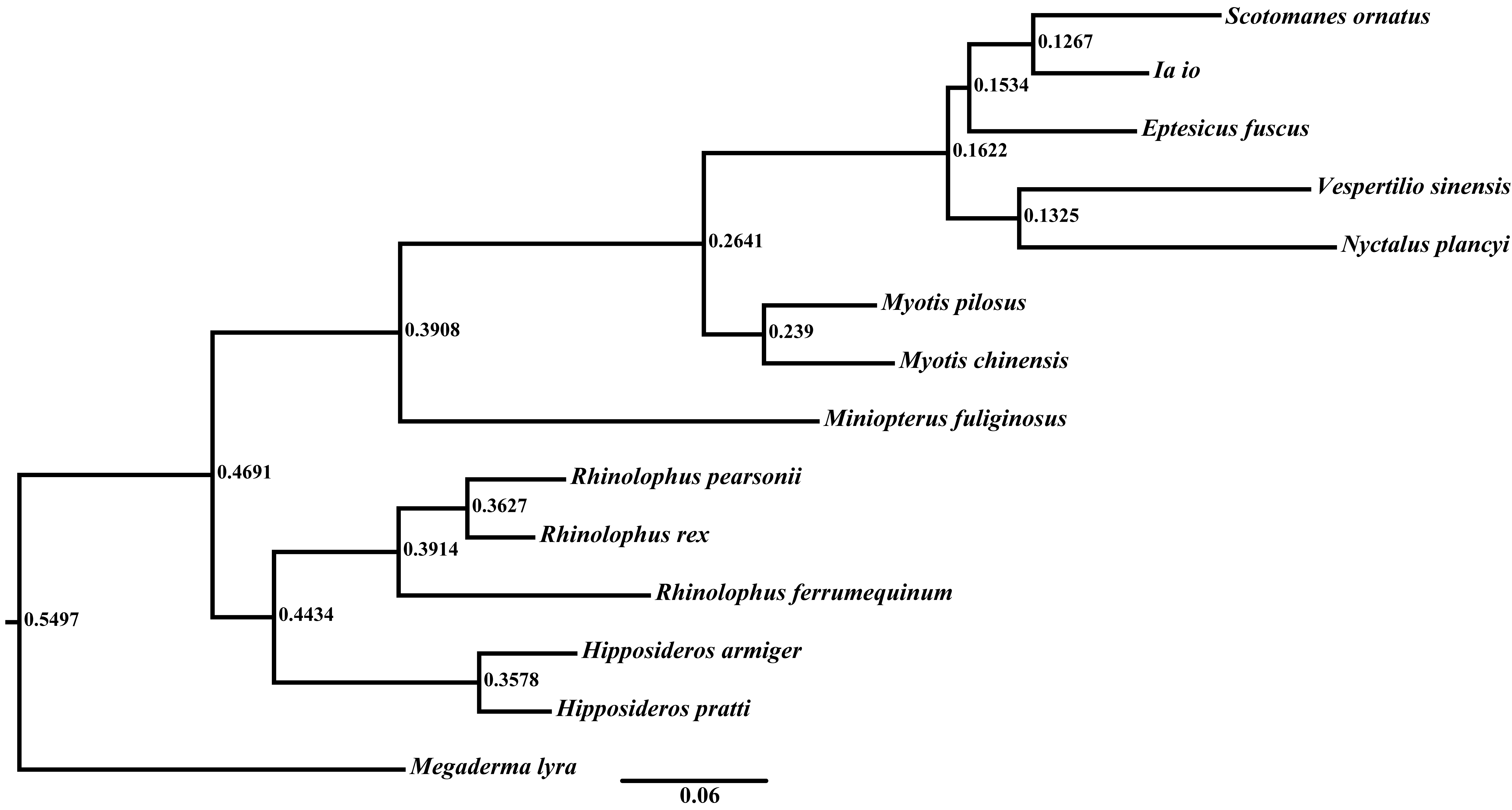

Supplement: Supplementary file 8 — Additional file 8 : Figure S4. Phylogenetic relationships among 14 species. [file 12983_2020_354_MOESM8_ESM.tif]
