# Supplementary material for: Correlation of skull morphology and bite force in a bird-eating bat (Ia io; Vespertilionidae)
Source: Front Zool. 2020 Mar 19;17:8. doi: 10.1186/s12983-020-00354-0 (PMC7082990; doi:10.1186/s12983-020-00354-0)
Supplement: Supplementary file 2 — Additional file 2 : Table S1. Paired comparison of skull morphological disparity with and without phylogenetic effects. [file 12983_2020_354_MOESM2_ESM.docx]

**Table S1.** Paired comparison (four diet categories) of skull morphological disparity with and without phylogenetic effects. PPV: Pairwise absolute differences between Procrustes variances. Black and bold fonts indicate significant differences between the two groups (*P*< 0.05).

| **group** | **Location** | **phylogeny** | | **nonphylogeny** | |
| --- | --- | --- | --- | --- | --- |
|  |  | **PPV** | ***P*-value** | **PPV** | ***P*-value** |
| **Bird-eating VS Carnivorous** | Mandible | < 0.0001 | 0.98 | 0.01142 | 0.097 |
|  | Dorsal | < 0.0001 | 1^a^ | 0.01225 | 1^a^ |
|  | Lateral | < 0.0001 | 1^a^ | 0.00939 | 1^a^ |
|  | Ventral | < 0.0001 | 1^a^ | 0.01916 | 0.717^a^ |
| **Bird-eating VS Insectivorous** | Mandible | 0.00818 | 0.065 | 0.00207 | 0.778 |
|  | Dorsal | 0.0119 | 0.201^a^ | 0.00166 | 1^a^ |
|  | Lateral | 0.01083 | 0.33^a^ | 0.00322 | 1^a^ |
|  | Ventral | 0.01553 | **0.018^a^** | 0.00108 | 1^a^ |
| **Bird-eating VS Piscivorous** | Mandible | < 0.0001 | 0.978 | 0.00327 | 0.685 |
|  | Dorsal | < 0.0001 | 1^a^ | 0.00556 | 1^a^ |
|  | Lateral | < 0.0001 | 1^a^ | 0.00203 | 1^a^ |
|  | Ventral | < 0.0001 | 1^a^ | 0.00105 | 1^a^ |
| **Insectivorous VS Piscivorous** | Mandible | 0.00818 | 0.076 | 0.0012 | 0.806 |
|  | Dorsal | 0.0119 | 0.21^a^ | 0.00726 | 1^a^ |
|  | Lateral | 0.01083 | 0.27^a^ | 0.00525 | 1^a^ |
|  | Ventral | 0.01553 | **0.018^a^** | 0.00003 | 1^a^ |
| **Insectivorous VS Carnivorous** | Mandible | 0.00818 | 0.079 | 0.00935 | 0.104 |
|  | Dorsal | 0.0119 | 0.246^a^ | 0.01395 | 0.531^a^ |
|  | Lateral | 0.01083 | 0.33^a^ | 0.01262 | 0.81^a^ |
|  | Ventral | 0.01553 | **0.018^a^** | 0.01808 | 0.342^a^ |
| **Carnivorous VS Piscivorous** | Mandible | < 0.0001 | 0.995 | 0.00815 | 0.299 |
|  | Dorsal | < 0.0001 | 1^a^ | 0.00670 | 1^a^ |
|  | Lateral | < 0.0001 | 1^a^ | 0.00737 | 1^a^ |
|  | Ventral | < 0.0001 | 1^a^ | 0.01811 | 0.795^a^ |

a, Bonferroni correction
