# Supplementary material for: Correlation of skull morphology and bite force in a bird-eating bat (Ia io; Vespertilionidae)
Source: Front Zool. 2020 Mar 19;17:8. doi: 10.1186/s12983-020-00354-0 (PMC7082990; doi:10.1186/s12983-020-00354-0)
Supplement: Supplementary file 5 — Additional file 5 : Table S2. Species, sample size (N), diets, and collection locations of bats. [file 12983_2020_354_MOESM5_ESM.docx]

**Table S2.** Species, sample size (N), diets and locations of bats.

| **Species** | **Bite force (N)** | **Cranium (N)** | **Mandible (N)** | **Diets** | **Locality** | **Longitude** | **Latitude** |
| --- | --- | --- | --- | --- | --- | --- | --- |
| *Ia io*^a^ | 21 | 6 | 6 | Bird-eating | Xingyi (GZ) | 104°52'43"E | 24°58'23"N |
| *Scotomanes ornatus*^a^ | 2 | 4 | 4 | Insectivorous | Chongyi (JX) | 114°12'49"E | 26°36'14"N |
| *Eptesicus fuscu*^a^ | 4 | 6 | 6 | Insectivorous | Jinning (YN) | 102°22'0"E | 24°29'59"N |
| *Nyctalus plancyi*^a^ | 10 | 3 | 3 | Insectivorous | Nanchong (SC) | 106°4'8"E | 30°49'13"N |
| *Vespertilio sinensis*^a^ | 19 | 5 | 5 | Insectivorous | Acheng (HLJ) | 126°57'26"E | 45°32'52"N |
| *Miniopterus fuliginosus*^a^ | 14 | 5 | 5 | Insectivorous | Xingyi (GZ) | 104°52'43"E | 24°58'23"N |
| *Myotis chinensis*^a^ | 25 | 5 | 5 | Insectivorous | Xingyi (GZ) | 104°52'43"E | 24°58'23"N |
| *Myotis pilosus*^a^ | 27 | 5 | 5 | Piscivorous | Xingyi (GZ) | 104°52'43"E | 24°58'23"N |
| *Hipposideros armiger*^b^ | 29 | 5 | 5 | Insectivorous | Xingyi (GZ) | 104°52'43"E | 24°58'23"N |
| *Hipposideros pratti*^b^ | 21 | 5 | 5 | Insectivorous | Xingyi (GZ) | 104°52'43"E | 24°58'23"N |
| *Rhinolophus ferrumequinum*^c^ | 17 | 5 | 5 | Insectivorous | Xingyi (GZ) | 104°52'43"E | 24°58'23"N |
| *Rhinolophus pearsonii*^c^ | 9 | 5 | 5 | Insectivorous | Xingyi (GZ) | 104°52'43"E | 24°58'23"N |
| *Rhinolophus rex*^c^ | 5 | 4 | 4 | Insectivorous | Xingyi (GZ) | 104°52'43"E | 24°58'23"N |
| *Megaderma lyra*^d^ | 1 | 6 | 6 | Carnivorous | Xingan (GX) | 110°40'42" E | 25°23'36"N |

a: Vespertilionidae; b: Hipposideridae; c: Rhinolophidae; d: Megadermatidae. GZ, Guizhou; JX, Jiangxi; YN, Yunnan; SC, Sichuan; HLJ, Heilongjiang; GX, Guangxi
