# Supplementary material for: Correlation of skull morphology and bite force in a bird-eating bat (Ia io; Vespertilionidae)
Source: Front Zool. 2020 Mar 19;17:8. doi: 10.1186/s12983-020-00354-0 (PMC7082990; doi:10.1186/s12983-020-00354-0)
Supplement: Supplementary file 6 — Additional file 6 : Description of the cranial and mandibular landmarks and semi-landmarks. [file 12983_2020_354_MOESM6_ESM.docx]

**Appendix S1.** Description of the cranial and mandibular landmarks and semi-landmarks

1. **Dorsal cranium**


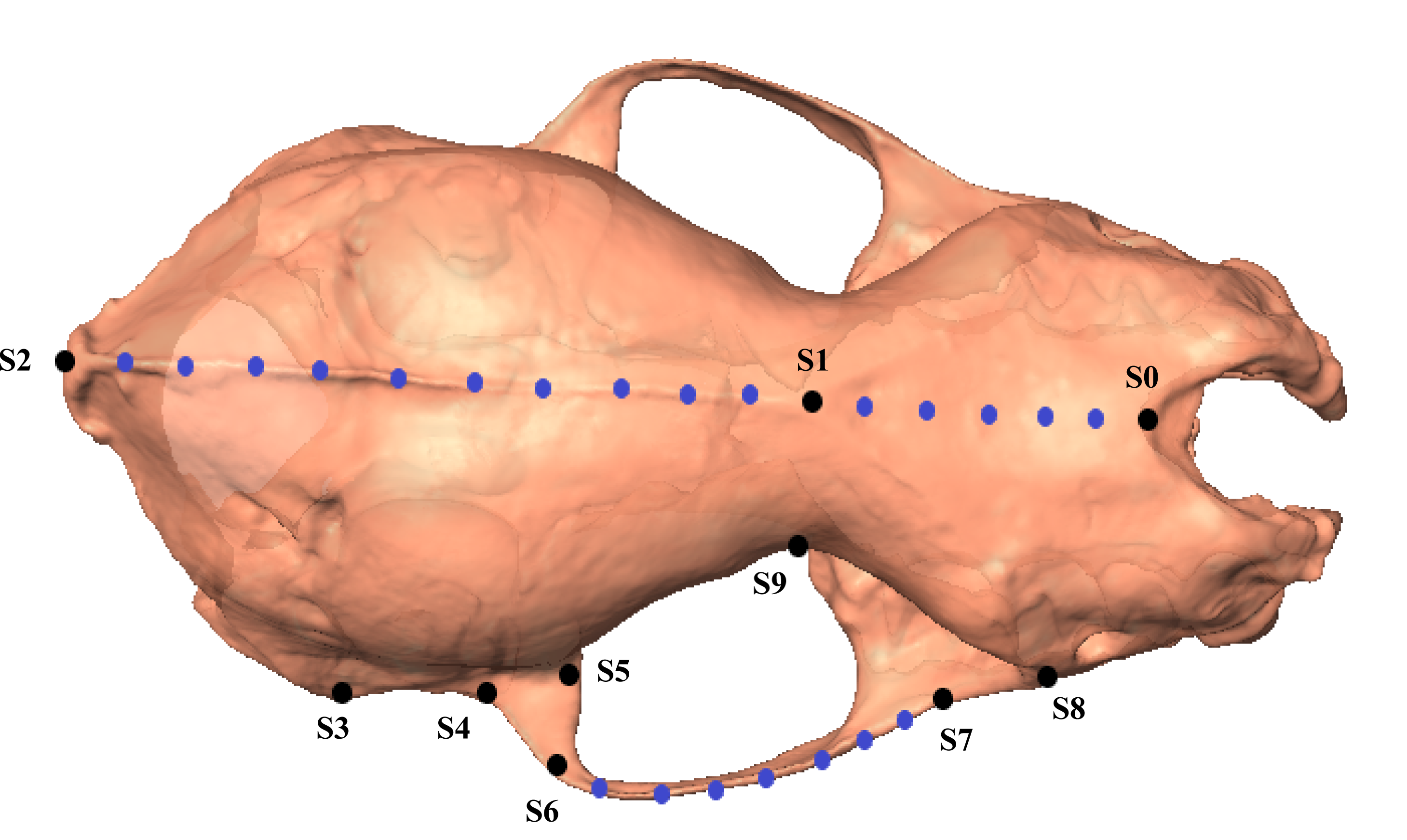


**Landmarks：**

S0: Midpoint tip of nasal

S1: The junction of the snout and frontal bone

S2: Most posterior point of the skull at the sagittal and lambdoidal crests

S3: Most lateral point of the mastoid process

S4: Point of insertion of the zygomatic arch on the braincase

S5: Point of insertion of the medial zygomatic arch on the braincase

S6: Most lateral point of the glenoid cavity

S7: Most dorsal point of the insertion of the jugal on the maxilla

S8: Most anterior point on the inflection of the orbit

S9: Narrowest point between frontals

**Semi-landmarks:**

C1: From S0 to S1 (n=5); C2: From S1 to S2 (n=10); C3: From S6 to S7 (n=7)

1. **Lateral cranium**

**
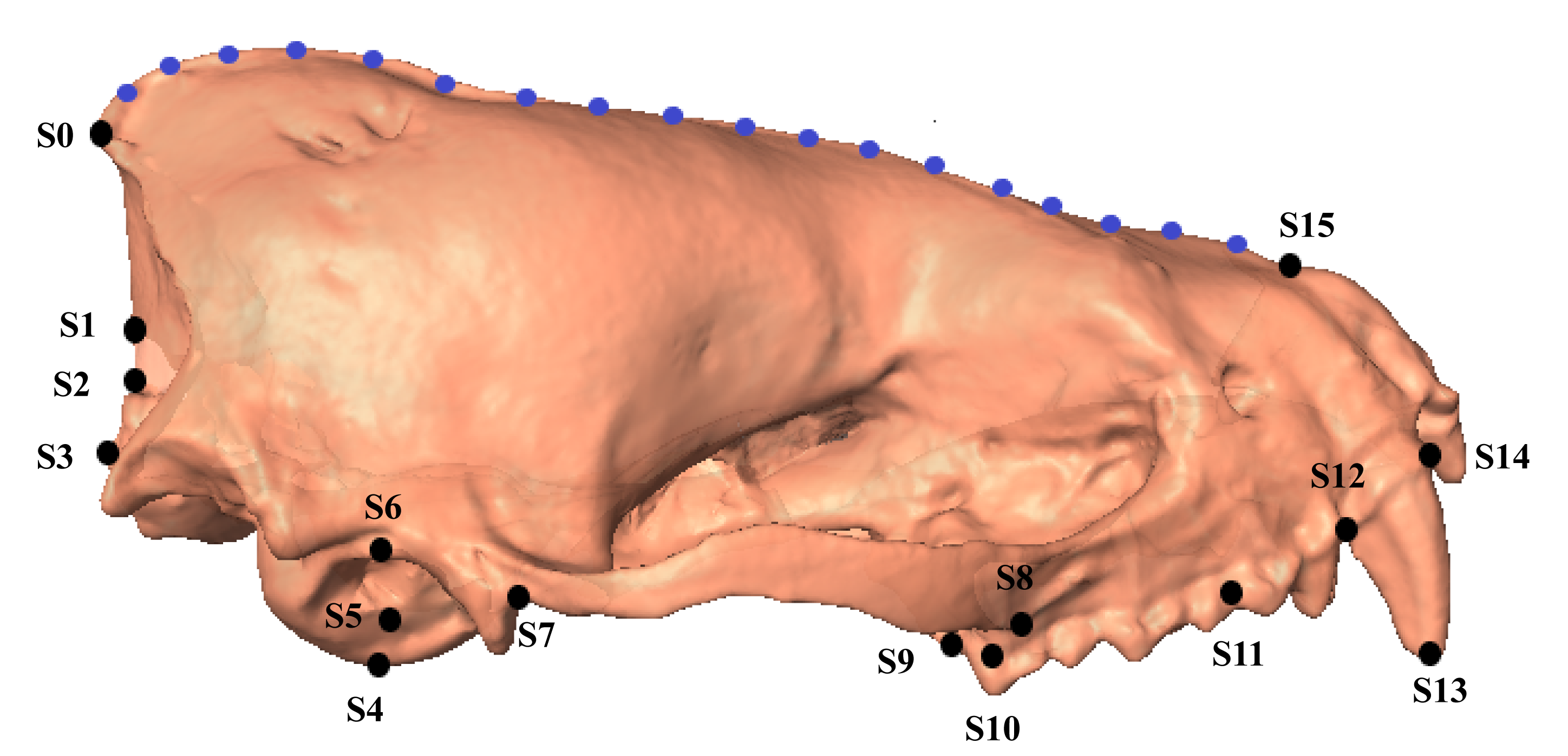
**

**Landmarks：**

S0: Most posterior point of the skull at the sagittal and lambdoidal crests

S1: Most ventral point of the supraoccipital

S2: The most curved point of the occipital fossa

S3: Most ventral and posterior point of the occipital bone

S4: The lowest point of the midline of the auditory bubble

S5: Most ventral point of the external auditory meatus

S6: Most dorsal point of the external auditory meatus

S7: Most lateral point on the margin of the mandibular fossa

S8: Most lateral point of the insertion of the jugal on the maxilla

S9: Most posterior point of the tooth row, at the base of the tooth

S10: Most posterior point on the last molar alveolus

S11: Most posterior point of the second premolar, at the base of the tooth

S12: Most posterior point of the canine, at the base of the tooth

S13: The point at the apex of a canine

S14: Most anterior point of the canine, at the base of the tooth

S15: Midpoint tip of nasal

**Semi-landmarks：**

C1: From S0 to S15 (n=18)

1. **Ventral cranium**

**
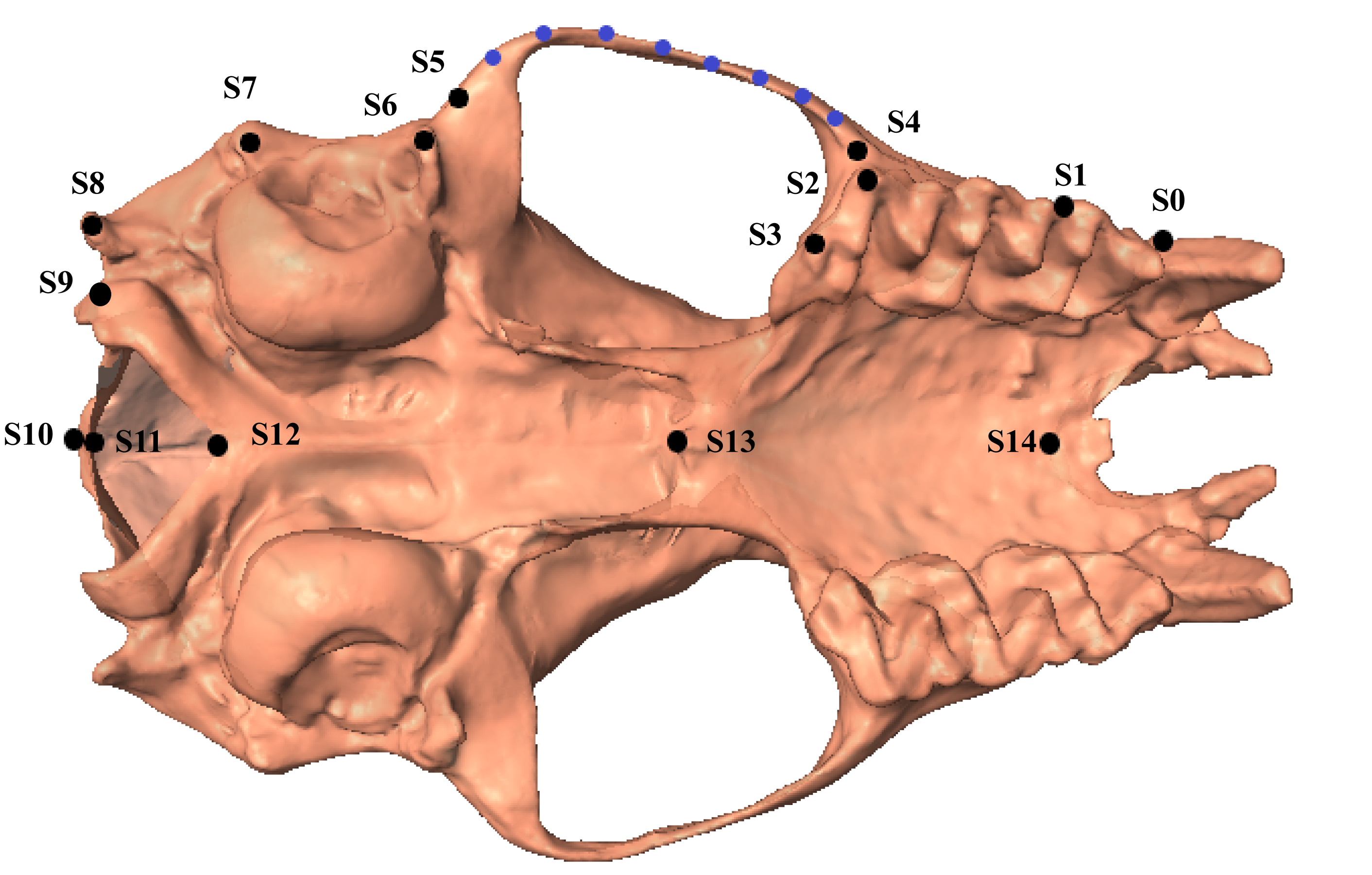
**

**Landmarks:**

S0: Most posterior and lateral point of the canine

S1: Most posterior marginal point of the second premolar

S2: Most posterior marginal point of the last molar alveolus

S3: Most posterior point of the tooth row

S4: The ventral intersection of the zygomatic anterior and the dentate bone

S5: Most lateral point on the margin of the mandibular fossa

S6: Anterior edge of the external auditory meatus

S7: Posterior edge of the external auditory meatus

S8: Tip of paraoccipital process

S9: Most lateral point of the occipital condyle

S10: Most posterior point of the cranium

S11: Posterior margin of the foramen magnum

S12: Anterior margin of the foramen magnum

S13: Most posterior point in the middle of the palatine

S14: Incisions of the premaxilla

**Semi-landmarks:** C1: From S4 to S5 (n=8)

1. **Mandibule**

**
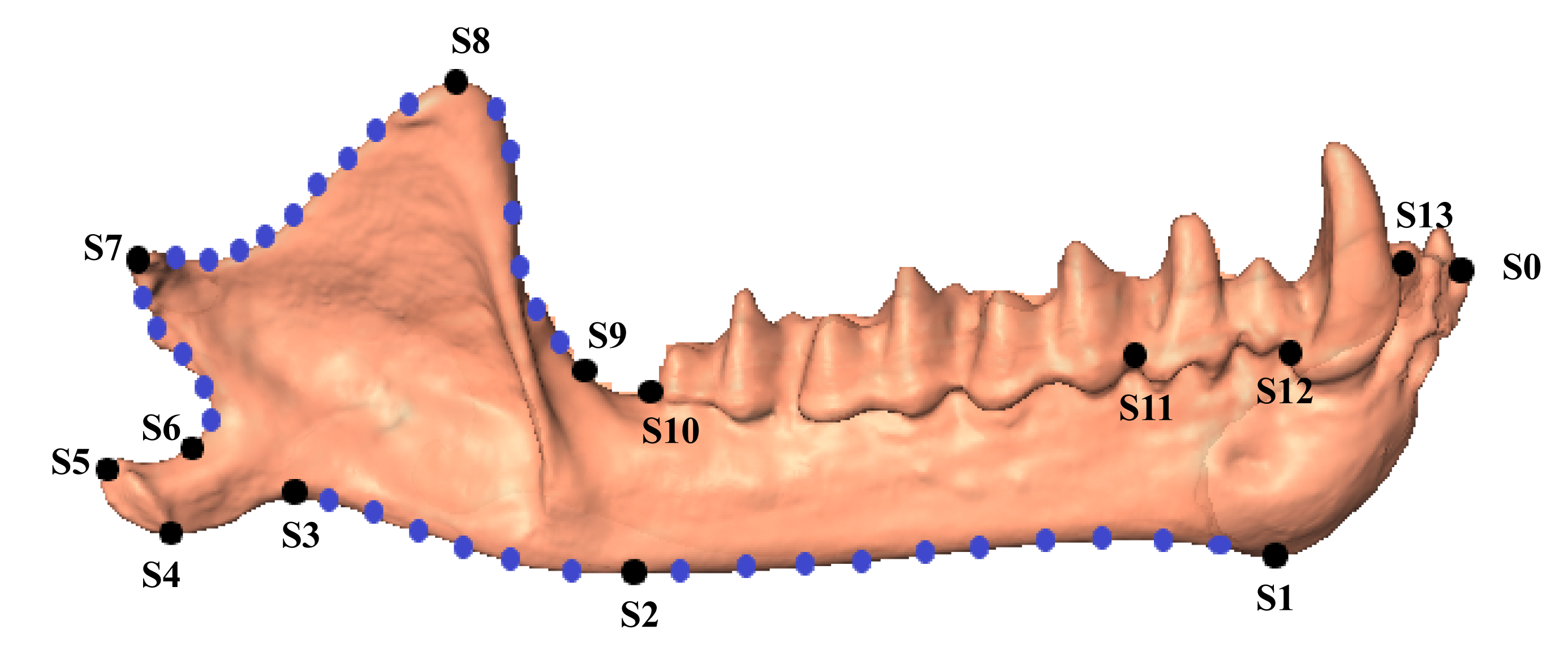
**

**Landmarks:**

S0: Most anterior point on the mandibular symphysis

S1: Anterior lower border of the mandible

S2: Most anterior point on the baseline perpendicular to the S10

S3: The inflection point between the ventral mandible and the angular process

S4: Posterolateral border of angular process

S5: Most posterior point on the angular process

S6: Inflection point on the posterior profile between the mandibular condyle and the angular process

S7: Most lateral point on mandibular condyle

S8: Most lateral extension of the coronoid process

S9: Anterolateral margin of the ramus

S10: Most posterior point on the last molar, at the base of the tooth

S11: Most posterior point of the second premolar, at the base of the tooth

S12: Most posterior point of the canine, at the base of the tooth

S13: Most anterior point of the canine, at the base of the tooth

**Semi-landmarks:**

C1: From S0 to S1 (n=10); C2: From S2 to S3 (n=6); C3: From S6 to S7 (n=5); C4: From S7 to S8 (n=9); C5: From S8 to S9 (n=6)
