# Supplementary material for: Correlation of skull morphology and bite force in a bird-eating bat (Ia io; Vespertilionidae)
Source: Front Zool. 2020 Mar 19;17:8. doi: 10.1186/s12983-020-00354-0 (PMC7082990; doi:10.1186/s12983-020-00354-0)
Supplement: Supplementary file 7 — Additional file 7 : Table S3. Information related to genes used in the study. [file 12983_2020_354_MOESM7_ESM.docx]

**Table S3.** Information for genes used in the study

| **Marker** | **Primer** | **Sequence**  **(5′-3′)** | **Annealing temperature**  **Ta( ℃)** | **Length (bp)** | **Mod** |
| --- | --- | --- | --- | --- | --- |
| **Cytb** | L14724 | CGAAGCTTGATATGAAAAACCATCGTTG | 44 | 1140 | TVMef + G;  HKY+ I+G;  HKY + I+G |
|  | H15915 | AACTGCAGTCATCTCCGGTTTACAAGAC |  |  |  |
| **Chd1** | EX26F | GATAARTCAGARACAGACCTTAGACG | 57 | 595 | TVM + G |
|  | EX27R | TTTGGCATTCACCTGYACTCC |  |  |  |
|  | EMB26F | TCAGGA ACAGAACGGACAGG |  |  |  |
|  | EMB27R | CACTTTCCCRAGTCTACCACCTTA |  |  |  |
| **Acox2** | ACOX2-F1 | CCTSGGCTCDGAGGAGCAGAT | 63 | 560 | HKY + I |
|  | ACOX2-R1 | GGGCTGTGHAYCACAAACTCCT |  |  |  |
